# Uso do Sistema de Informação de Imunização do Brasil: qual a realidade?**

**DOI:** 10.15649/cuidarte.2138

**Published:** 2022-08-20

**Authors:** Samuel Barroso Rodrigues, Gabriela Gonçalves Amaral, Brener Santos Silva, Gabriela Cunha Corrêa Freitas de Oliveira, Laís Oliveira de Moraes Tavares, Valéria Conceição de Oliveira, Eliete Albano de Azevedo Guimarães

**Affiliations:** 1 Universidade Federal de São João del-Rei (UFSJ/CCO). Divinópolis, MG, Brasil. Email: samuelbarroso88@gmail.com Universidade Federal de São João del-Rei Universidade Federal de São João del-Rei Divinópolis MG Brazil samuelbarroso88@gmail.com; 2 Universidade de São Paulo (EERP/ USP). Ribeirão Preto, SP, Brasil. Email: g.amaral@usp.br Universidade de São Paulo Universidade de São Paulo Ribeirão Preto SP Brazil g.amaral@usp.br; 3 Universidade de São Paulo (EERP/ USP). Ribeirão Preto, SP, Brasil. Email: brenersantos@usp.br Universidade de São Paulo Universidade de São Paulo Ribeirão Preto SP Brazil brenersantos@usp.br; 4 Universidade Federal de São João del-Rei (UFSJ/CCO). Divinópolis, MG, Brasil. E-mail: gabyccunha@gmail.com Universidade Federal de São João del-Rei Universidade Federal de São João del-Rei Divinópolis MG Brazil gabyccunha@gmail.com; 5 Universidade Federal de São João del-Rei (UFSJ/CCO). Divinópolis, MG, Brasil. Email: laisoliveiramt@gmail.com Universidade Federal de São João del-Rei Universidade Federal de São João del-Rei Divinópolis MG Brazil laisoliveiramt@gmail.com; 6 Universidade Federal de São João del-Rei (UFSJ/CCO). Divinópolis, MG, Brasil. Email: valeriaoliveira@ufsj.edu.br Universidade Federal de São João del-Rei Universidade Federal de São João del-Rei Divinópolis MG Brazil valeriaoliveira@ufsj.edu.br; 7 Universidade Federal de São João del-Rei (UFSJ/CCO). Divinópolis, MG, Brasil. Email: elietealbano@ufsj.edu.br Autor de correspondência Universidade Federal de São João del-Rei Universidade Federal de São João del-Rei Divinópolis MG Brazil elietealbano@ufsj.edu.br

**Keywords:** Programas de Imunização, Sistemas de Informação, Avaliação em Saúde, Atenção Primária à Saúde, Enfermagem em Saúde Pública, Immunization Programs, Information Systems, Health Evaluation, Primary Health Care, Public Health Nursing, Programas de Inmunización, Sistemas de Información, Evaluación en Salud, Atención Primaria de Salud, Enfermería en Salud Pública

## Abstract

**Introdução:**

A informação em saúde é essencial na tomada de decisões no âmbito das políticas públicas e tem se apresentado como instrumento essencial na interpretação de fenômenos. Assim, oestudo teve comoobjetivo avaliar o uso do Sistema de Informação de Imunização pelos profissionais de enfermagem.

**Materiais e métodos:**

Estudo transversal analítico realizado num município da Macrorregião Oeste de Minas Gerais, com profissionais de enfermagem por meio de um *checklist* validado. Para análise, utilizou-se um sistema de escores, classificado como: adequado, parcialmente adequado, não adequado e crítico. A mediana foi utilizada como medida-resumo para a análise descritiva e o Teste Qui-Quadrado de Pearson, para comparação de proporções.}

**Resultados:**

Dos 104 profissionais de enfermagem, 14,4% relatam fazer uso dos registros para a gestão da informação em vacinação, 93,3% realizam o controle de estoque dos imunobiológicos, sendo esta a única atividade classificada como adequada. A produção do relatório de listagem de faltosos (39,4%), produção do relatório de cobertura vacinal (36,5%) e divulgação das informações consolidadas produzidas (17,3%) foram classificados como críticos. Não houve associações estatisticamente significativas entre as variáveis estudadas.

**Discussão:**

A subutilização das informações do sistema de informação traz consequências para os serviços de imunização, como baixas coberturas vacinais e oscilações nas proporções de abandono, além dificultar o planejamento e a tomada de decisões dos gestores das salas de vacinação.

**Conclusões:**

O uso das informações dos sistemas de informação precisa ser reconhecido pelos profissionais como necessário, útil e aplicável, sendo parte do processo de trabalho em sala de vacinação.

## Abstract (en)

**Introduction:**

Public health information is essential for decision-making in the public policy context, therefore considered a basic tool for phenomenon interpretation. The objective of this study was to evaluate the use of the Immunization Information System by nursing professionals.

**Materials and Methods:**

An analytical cross-sectional study was conducted with nursing professionals in a municipality in the Western Macro-region of Minas Gerais State by means of a validated checklist. For analysis, a scoring system was used to rate their use of the information system as appropriate, partially appropriate, not appropriate and critical. The median was used as a summary measure for descriptive analysis and Pearson’s chi-square test was used to assess comparisons.

**Results:**

Out of 104 participating nurses, 14.4% reported having used records for vaccination information management; 93.3% performed vaccine inventory management, which was the only activity classified as appropriate; preparation of reports listing missed doses (39.4%), vaccination coverage (36.5%) and dissemination of consolidated reports (17.3%) were classified as critical. No statistically significant differences were found between the variables studied.

**Discussion:**

Underutilizing information systems has consequences for vaccination services, such as low vaccination coverage and variation in dropout rates, as well as hindering planning and decision making by vaccination center managers.

**Conclusions:**

Managing data in information systems should be recognized by healthcare professionals as a necessary, functional and applicable piece of the work process performed in vaccination centers.

## Resumen

**Introducción:**

La información sanitaria es esencial para la toma de decisiones en el contexto de políticas públicas, por lo que se considera como una herramienta básica en la interpretación de los fenómenos. El objetivo de este estudio fue evaluar el manejo del Sistema de Información de Inmunización por parte de los profesionales de enfermería.

**Materiales y métodos:**

Estudio transversal analítico realizado en un municipio de la Macrorregión Oeste de Minas Gerais con profesionales de enfermería mediante una lista de verificación validada. Para el análisis, se utilizó un sistema de puntuación para clasificar el manejo del sistema como adecuado, parcialmente adecuado, no adecuado y crítico. Se utilizó la mediana como medida de resumen para el análisis descriptivo y la prueba de chi-cuadrado de Pearson para comparar proporciones.

**Resultados:**

De los 104 profesionales de enfermería participantes, el 14.4% afirmó haber usado los registros en la gestión de la información en vacunación; el 93.3% realiza control de las existencias de inmunobiológicos, siendo esta la única actividad clasificada como adecuada; la elaboración de informes con la lista de dosis perdidas (39.4%), la cobertura de vacunación (36.5%) y la divulgación de informes consolidados (17.3%) se clasificaron como crítico. No se encontraron asociaciones estadísticamente significativas entre las variables estudiadas.

**Discusión:**

La subutilización del sistema de información trae consecuencias para los servicios de vacunación, como baja cobertura de vacunación y las variaciones en las tasas de abandono, además de generar dificultades en la planificación y en la toma de decisiones por parte de los gestores en las salas de vacunación.

**Conclusiones:**

El manejo de la información que reposa en los sistemas de información debe reconocerse por parte de los profesionales de la salud como necesario, útil y aplicable, por lo que debe hacer parte del proceso del trabajo que se realiza en las salas de vacunación.

## Introdução

A informação em saúde é essencial na tomada de decisões no âmbito das políticas públicas e tem se apresentado como instrumento essencial na interpretação de fenômenos, o que gera a perceptibilidade de significados antes invisíveis^1^. Embora tenha papel fundamental na gestão em saúde desde o século XIX, a informação sofreu modificações ao longo dos anos, sobretudo no que se refere à sua forma de extração, armazenamento e disseminação pelos canais ou sistemas de rede, incluindo os sistemas de informação em saúde (SIS)^2^.

Os SIS, considerados inovações tecnológicas, foram criados com a finalidade de disseminar a informação e instrumentalizar a coleta, análise e o processamento dos dados, potencializando as ações estratégicas em saúde^3^^,^^4^. Dentre os SIS, os sistemas de informação em imunização (SII), implantados na década de 1970 nos Estados Unidos, destacam-se por se apresentarem como confidenciais, confiáveis e de fácil acesso^5^. No Brasil, o SII utilizado recebe o nome de Sistema de Informação do Programa Nacional de Imunização (SIPNI), criado em 2010 e implantado pelo Programa Nacional de Imunizações (PNI) em 2014. Este sistema armazena informações de forma individual e coletiva, possibilitando a avaliação dessas informações e gerando conhecimentos para a gestão dos serviços de imunização^4^^,^^6^^-^^8^.

Os SII podem ser utilizados como estratégia de monitoramento das coberturas vacinais; incentivo a adesão às vacinas agendadas; identificação e atualização de esquemas vacinais em atraso, assim como a verificação das vacinas em idade recomendada e vigilância de eventos adversos pós-vacinação (EAPV)^5^^,^^9^^-^^11^. Portanto, tais sistemas são concebidos como ações estratégicas que influenciam diretamente na tomada de decisões e potencializam a ação dos gestores, além de subsidiar o planejamento de políticas públicas e o fortalecimento das ações dos PNI^12^.

Estudos nacionais e internacionais sobre SII apontam que os registros de imunização oportunizam o monitoramento da cobertura vacinal, o fornecimento de dados sobre EAPV, o controle e validade dos imunobiológicos^8^^,^^11^^,^^13^^-^^16^ e melhora a tomada de decisões frente às atividades de vacinação. As evidências na literatura brasileira apontam que os registros de imunização são na sua maioria utilizados para fins de pesquisas^10^^,^^17^. Contudo, ainda são escassas publicações que abordam o uso desses registros pelos profissionais de saúde para monitorar e avaliar os serviços de imunização.

A proposta deste estudo avança no conhecimento, ao investigar se há gestão do SII pela equipe de enfermagem das unidades de atenção primária à saúde (UAPS). Assim, este estudo buscou avaliar o uso do Sistema de Informação de Imunização pelos profissionais de enfermagem.

## Materiais e métodos

Trata-se de um estudo transversal analítico realizado nas salas de vacinação das UAPS de um município de grande porte da Macrorregião Oeste de Minas Gerais, entre os meses de janeiro e junho de 2019. O município possui 43 UAPS na zona urbana, destas, 32 são Estratégia de Saúde da Família e 11 Unidades de Saúde Tradicional, distribuídas em 10 regiões sanitárias. Todas foram incluídas no estudo. Ressalta-se que todas as UAPS possuíam salas de vacinação e que no ano de 2019 foram administradas 37.441 doses de vacinas^18^.

Foram incluídos na pesquisa profissionais de enfermagem (enfermeiros, técnicos de enfermagem e auxiliares de enfermagem) atuantes nas salas de vacinação com SII implantado, com pelo menos seis meses de atuação em sala de vacinação (devido ao fato de ter um conhecimento mais amplo acerca da rotina de trabalho) e que manifestasse interesse em participar do estudo. Após o convite, sua participação foi formalizada pela assinatura do Termo de Consentimento Livre e Esclarecido. Excluiu-se aqueles profissionais ausentes nas UAPS na data da coleta de dados (com atestado médico ou em regime de férias). Dos 219 profissionais de enfermagem elegíveis, 104 participaram do estudo.

Para a coleta de dados foi utilizado o *Checklist* para Avaliação do Sistema de Informação de Imunização (CASII), instrumento validado no contexto brasileiro para avaliar o SII do Brasil nas salas de vacinação^19^. O *checklist* é composto por variáveis sociodemográficas e laborais dos profissionais, autoavaliação do conhecimento no SII e variáveis acerca do SII, estas estratificadas em duas dimensões estrutura e processo, esta última contendo 3 componentes: gestão do SII; registro do vacinado e movimento dos imunobiológicos^19^ (material suplementar).

Inicialmente, realizou-se a análise descritiva dos dados utilizando-se de medidas de frequência para todas as variáveis de estudo. A medida de posição utilizada para as variáveis sociodemográficas e laborais foi a mediana, adequada para a análise de distribuições não paramétricas.

Para avaliar o uso das informações do SII, foi utilizada uma matriz de análise contendo somente as variáveis da dimensão processo. Entende-se que as variáveis estruturais podem influenciar no uso das informações do SII, mas é o processo que se refere as atividades realizadas (serviços e bens produzidos)^7^.

Para cada variável de processo foi definido o valor máximo atribuído de pontos e calculada a média de respostas dos participantes em relação a esse valor. A cada pergunta, o profissional pôde escolher entre as opções: sim = 10 pontos; às vezes = 5 pontos e não = zero pontos. Os escores obtidos a partir da soma dos pontos das variáveis do componente foram transformados em percentuais, com referência à pontuação máxima possível (Σ observados/Σ dos pontos máximos esperados X 100). A partir desses percentuais, foram definidas as categorias para o uso da informação em quatro estratos: a) adequado = ≥ a 80%; b) parcialmente adequado = 60% a 79,9%; c) não adequado = 40% a 59,9%; e d) crítico = < a 40%.

Para verificar a associação das variáveis sociodemográficas e laborais que influenciavam o uso da informação do SII utilizou-se o Teste do Qui-Quadrado de Pearson, com nível de significância de 5%. Os dados^20^ foram processados no *software Excel* (versão 2015) e analisados no *software Statistical Package for Social Sciences* (versão 21.0). O estudo recebeu aprovação pelo Comitê de Ética em Pesquisa da Universidade Federal de São João del-Rei, Campus Centro-Oeste Dona Lindu sob o CAAE 65656017.6.0000.5545 e parecer no 3.354.176.

## Resultados

Entre os profissionais de enfermagem, a maioria era do sexo feminino (82; 78,8%), com idade entre 28 a 63 anos (mediana = 39 anos) e com tempo de formação entre 2 e 40 anos (mediana=15 anos). Dos participantes, 66 (63,5%) eram técnicos de enfermagem e 38 (36,5%) enfermeiros e o tempo de trabalho em sala de vacinação teve uma mediana de 8 anos. Quanto à autoavaliação do conhecimento sobre o SII, 85,6% dos entrevistados declararam o conhecimento igual ou superior a seis.

Na dimensão estrutura observou-se que a maioria dos profissionais (101; 97,1%) relatou trabalhar em salas de vacinação com computador para uso do SII, contendo impressora e insumos básicos para impressão. O município disponibiliza um técnico de informática para suporte informacional e um canal de comunicação para esclarecimentos de dúvidas, contudo uma parte dos profissionais relatou não conhecer nenhum destes canais (15; 14,4%). Apesar de 82,7% dos profissionais terem sido capacitados para operacionalizar o SII, 90,4% relataram não receber capacitações para a gestão do sistema. Quanto à conectividade mais da metade dos profissionais (68; 65,4%) relataram ter internet estável. Ademais, quase totalidade dos profissionais (98; 94,2%) relatou que os mesmos dados registrados no SII são também registrados em papel, configurando-se em um retrabalho e dispendioso gasto de tempo (Tabela 1).

Na dimensão processo, observou-se que a maioria dos profissionais não emitem relatórios de coberturas vacinais, somente 40,4% usam informações do SII para o cálculo da taxa de abandono e 52,9% tem cadastro das pessoas da área de abrangência. Destaca-se que a conferência de doses registradas no SII com a caderneta de vacinação do usuário foi relatada por quase totalidade dos profissionais (Tabela 1).

**Tabela 1:** Caracterização das variáveis estruturais e de processo do Sistema de Informação de Imunização, em um município de Minas Gerais, 2019 (n = 104).

Dimensão Estrutura	Sim n (%)
Existência de computador com SII instalado	101 (97,1)
Existência de mobiliários básicos para uso do SII	101 (97,1)
Existência de impressora disponível para uso dos profissionais	101 (97,1)
Existência de insumos básicos para impressão	82 (78,8)
Existência de manualdo SII para consulta do profissional	68 (65,4)
Existência de profissional técnico para suporte ao SII	103 (99,0)
Uso de canal de comunicação para esclarecimento de dúvidas	78 (75,0)
Capacitação para operacionalizar o SII	86 (82,7)
Capacitação para agestão do SII	10 (9,6)
Acesso à internet estável na sala de vacinação	68 (65,4)
Manutenção dos registros de imunização manualmente/papel	98 (94,2)
Dimensão Processo	Sim n (%)
Cadastro das pessoas da área de abrangência da unidade de saúde no SII	55 (52,9)
Produção do relatório de doses aplicadas	81 (77,9)
Produção do relatório de listagem de faltosos	41 (39,4)
Produção do relatório de cobertura vacinal	38 (36,5)
Uso das informações do SII para controle de estoque	97 (93,3)
Uso das informações do SII para cálculo da taxa de abandono	42 (40,4)
Divulgação das informações geradas	18 (17,3)
Realização do aprazamento manual no sistema	58 (55,8)
Conferência de doses registradas no SII com a caderneta de vacinação do usuário	103 (99,0)

SII: Sistema de Informação em Imunização.

Fonte: Elaborado pelos autores, 2021.

**Figura 1:**
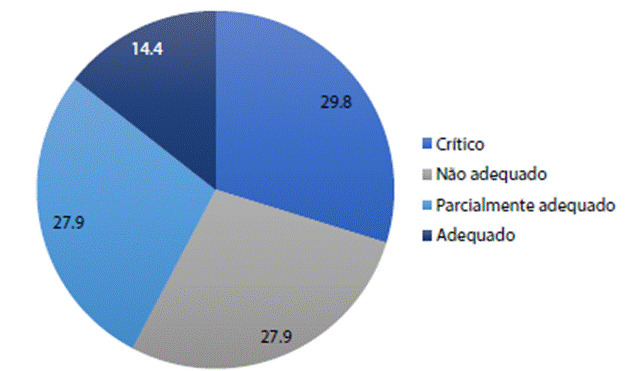
Classificação do grau de desempenho do uso das informações do Sistema de Informação de Imunização, em um município de Minas Gerais, 2019.

Ao analisar o uso dos registros de imunização do SII pelos profissionais de enfermagem identificou-se que este uso foi classificado em sua maioria como crítico (31; 29,8%), seguido de não adequado (29; 27,9%), parcialmente adequado (29; 27,9%) e adequado (15; 14,4%), demonstrando que uma pequena parcela de profissionais usa os registros de imunização adequadamente para a gestão da informação em vacinação (Figura 1).

Fonte: Elaborado pelos autores, 2021.

Com relação aos critérios utilizados para avaliar o uso das informações do SII, observou-se que o controle de estoque dos imunobiológicos (93,3%) foi a única atividade em que o uso da informação obteve pontuação adequada. O segundo critério melhor avaliado foi a produção de relatório de doses aplicadas (77,9%), que foi classificado como parcialmente adequado (Tabela 2). Sabe-se que estas atividades, que alcançaram as maiores pontuações, são de caráter obrigatório no SII.

**Tabela 2:** Uso das informações do Sistema de Informação de Imunização segundo variáveis da dimensão processo, em um município de Minas Gerais, 2019.

Dimensão processo	Máximo de pontos	Uso das **informações**	Classificação do GDa
Cadastro das pessoas da área de abrangência da unidade de saúde no SII	10	52,9	não adequado
Produção do relatório de doses aplicadas	10	77,9	Parcialmente adequado
Produção do relatório de listagem de faltosos	10	39,4	crítico
Produção do relatório de cobertura vacinal	10	36,5	crítico
Uso das informações para controle de estoque	10	93,3	adecuado
Uso das informações para cálculo da taxa de abandono	10	40,4	não adequado
Divulgação das informações geradas	5	17,3	crítico
Total	65	53,7	não adequado

SII: Sistema de Informação em Imunização.

Nota: aGD: Grau de Desempenho = Σ observados/ Σ dos pontos máximos esperados x 100.

Fonte: Elaborado pelos autores, 2021.

Outras atividades como a produção do relatório de listagem de faltosos (39,4%), produção do relatório de cobertura vacinal (36,5%) e divulgação das informações consolidadas produzidas no SIS (17,3%) alcançaram os piores escores, sendo classificados como uso crítico (Tabela 2). Ressalta-se que, se devidamente executadas, estas atividades contribuem potencialmente para o planejamento das ações dos profissionais de enfermagem atuantes em sala de vacinação, além de melhorar os indicadores de vacinação municipal.

Na análise bivariada, não se identificou associações estatisticamente significativas entre as variáveis sociodemográficas e laborais com o uso das informações do SII (Tabela 3).

**Tabela 3:** Associação entre as características dos profissionais de enfermagem com o grau de desempenho na gestão dos registros informatizados de imunização, em um município de Minas Gerais, 2019 (n = 104)

	Uso das informações do SII^a^				
Variáveis	Adequado	Parcialmente adequado	Não adequado	Crítico	pb
	n (%)	n (%)	n (%)	n (%)	
Categoria profissional					0,067
Enfermeiro	4 (10,5)	17 (44,7)	9 (23,7)	8 (21,1)	
Técnico de enfermagem	11 (19)	11 (19)	16 (27,6)	20 (34,5)	
Auxiliar de enfermagem	0 (0)	1 (12,5)	4 (50)	3 (37,5)	
Nível de formação					0,060
Ensino médio profissionalizante	11 (18,6)	11 (18,6)	16 (27,1)	21 (35,6)	
Ensino superior	4 (8,9)	18 (40)	13 (28,9)	10 (22,2)	
Autoavaliação do conhecimento do SII					0,073
< 6	4 (26,7)	2 (13,3)	7 (46,7)	2 (13,3)	
≥ 6	11 (12,4)	27 (30,3)	22 (24,7)	29 (32,6)	
Idade					0,647
< 30 anos	3 (23,1)	2 (15,4)	4 (30,8)	4 (30,8)	
≥ 30 anos	12 (13,2)	27 (29,7)	25 (27,5)	27 (29,7)	
Tempo de trabalho em sala de vacinação					0,432
< 5 anos	7 (21,2)	7 (21,2)	8 (24,2)	11 (33,3)	
≥ 5 anos	8 (11,3)	22 (31)	21 (29,6)	20 (28,2)	

SII: Sistema de Informação em Imunização.

Nota: aUso das informações do SII = Σ observados/ Σ dos pontos máximos esperados x 100); bTeste Qui-Quadrado de Pearson com nível de significância de 95%.

Fonte: Elaborado pelos autores, 2021.

## Discussão

Os profissionais de enfermagem, em sua maioria, não utilizam os dados do SII para a gestão das informações em sala de vacinação. E a subutilização desses dados não estão associados a categoria profissional, nível de formação, idade, tempo de trabalho em sala de vacinação e nível de conhecimento do SII.

Percebe-se que os SII, apesar de não apresentarem problemas estruturais de grande relevância, é subutilizado, o que acaba reduzindo a sua aplicabilidade, sobretudo no poder decisório efetivo desse sistema. Essa realidade mostra que a diminuição desse uso pode estar mais relacionada às tecnologias leves (as quais se referem às relações) do que às tecnologias duras (relacionadas aos recursos materiais)^21^.

Observou-se, no município estudado, a existência de capacitações para operacionalizar o sistema, contudo não foi relatado pelos profissionais a continuidade de treinamentos para a gestão do SII. A implantação do SII por si só não é suficiente para impactar na qualidade dos serviços prestados. É necessário que os dados produzidos sejam utilizados para a tomada de decisão nos serviços de imunização com o propósito de manter a eficiência dos serviços e a qualidade das funcionalidades do sistema.

De maneira geral, os elementos mais comprometedores no uso do SII estão relacionados ao processo, apontando uma necessidade de mudança das atividades desenvolvidas nas relações de produção entre profissionais e vacinados^22^. Neste estudo, ao analisar a dimensão processo, percebe-se que os indicadores avaliados ficaram abaixo dos estratos não adequado e crítico para o uso da informação. As ações que apresentaram melhor classificação do uso dos dados foram aquelas de caráter obrigatório, cujo teor é logístico para a sala de vacinação, como o registro do estoque de imunobiológicos e produção de relatórios de doses aplicadas.

Estudos relacionados à temática da avaliação das dimensões estrutura e processo do SII do Brasil ainda são incipientes no país. Outros estudos^8^^,^^23^ que utilizaram outras metodologias também evidenciaram entraves similares, principalmente relacionados a falta de registro individual do vacinado por local de residência, duplicidade de dados e subutilização do sistema informacional. Tais entraves podem impactar na subestimação da população, oscilação nas proporções de abandono e distorções na cobertura vacinal, fazendo com que o SII não reflita o real contexto atual^8^^,^^23^.

Os SII são capazes de oportunizar dados acurados e de proporcionar avaliação da cobertura vacinal e de EAPV, controle de estoque, validade das doses e verificar oportunidades de vacinação^10^^,^^11^^,^^16^^,^^24^^,^^25^. Essas informações, devidamente utilizadas, potencializam a qualidade da assistência prestada e, consequentemente, contribui para o sucesso do PNI brasileiro. Porém, o cadastro insuficiente da população adscrita, dados incipientes dos relatórios de cobertura vacinal e, de maneira ainda mais relevante, uma limitada divulgação dessas informações apontadas neste estudo, compromete a utilização do SII. Tal subutilização caminha na contramão do planejamento em saúde, uma vez que os indicadores de saúde gerados pelos SII se configuram como importante recurso de apoio aos gestores, sobretudo no direcionamento da tomada de decisões^26^.

Como forma de melhorar a completude dos dados no Sistema, bem como o uso das informações geradas por este, a adoção de capacitações com metodologias ativas, como a simulação realística^27^, aprimoramento da usabilidade do Sistema^28^^,^^29^ e melhorias na infraestrutura organizacional, como acesso ao computador e à internet de boa conectividade^23^, podem facilitar o manuseio do SII, diminuir a resistência dos profissionais de enfermagem quanto ao uso da tecnologia e melhoria na assistência e registro das informações de forma correta.

Apesar das vantagens do SII existe um distanciamento entre sua finalidade e a realidade do seu uso pelos serviços de imunização. O uso reduzido das informações pode ser justificado por diversos fatores, como divergências na qualidade e completude dos dados^17^^,^^26^; pouca integração e lentidão do Sistema^28^^,^^29^; falta de capacitação dos profissionais de saúde para a operacionalização e gestão do Sistema^22^^,^^23^^,^^30^ e número reduzido de profissionais^31^.

Em estudo realizado para avaliação da aceitação e uso do SIPNI, os profissionais atuantes em sala de vacinação perceberam que o SII disponibiliza dados em nível individual, inseridos em tempo real, o que oportuniza o monitoramento da cobertura vacinal^8^. Contudo a subutilização desses dados traz consequências para os serviços de imunização, como baixas coberturas vacinais e oscilações nas proporções de abandono^11^^,^^16^^,^^17^.

Estudo realizado em municípios de Minas Gerais identificou que mesmo após a implantação do SIPNI, ainda havia a manutenção dos registros em papel, sobrecarregando os profissionais com o retrabalho dos registros^23^. Tais resultados também foram apontados pelos entrevistados dessa pesquisa.

Uma das limitações compete à abordagem exclusivamente utilizada (quantitativa) que, apesar de ter respondido ao objetivo, não é suficientemente capaz de qualificar as variáveis de contexto que podem explicar os aspectos subjetivos que possam estar associados ao desfecho. Portanto, novas pesquisas de base qualitativa também devem ser realizadas a fim de se analisar o desempenho dos profissionais em relação ao uso dos SII. De qualquer modo, destaca-se a importância de diferentes estratégias metodológicas para a compreensão dos fatores impeditivos deste processo, a fim de que esta ferramenta possa revelar seu potencial em desenvolver e implementar a tomada de decisão nos serviços de imunização.

Como ponto forte, o estudo engendra uma visão geral do objeto de estudo, que é o uso das informações do SII pelos profissionais de enfermagem para a gestão da informação em vacinação. Os resultados oferecem uma análise ampliada acerca dos entraves e das facilidades no manuseio dos recursos informacionais do profissional que trabalha em salas de vacinação, servindo ainda de eixo norteador para novas pesquisas na área.

## Conclusões

O estudo aponta que há deficiências no uso das informações do SII pelos profissionais de enfermagem das UAPS. Os problemas identificados referem-se as atividades de produção de relatório de cobertura vacinal, listagem de faltosos, informações para cálculo de abandono, cadastro das pessoas da área de abrangência da unidade de saúde no SII e divulgação das informações.

O uso das informações do SII precisa ser reconhecido pelos profissionais como necessário, útil e aplicável, sendo parte do processo de trabalho em sala de vacinação. Assim, torna-se essencial estratégias de motivação, sensibilização, capacitação e supervisão para a utilização maximizada das informações de imunização no processo decisório.

## Figures and Tables

**Figura 2:**
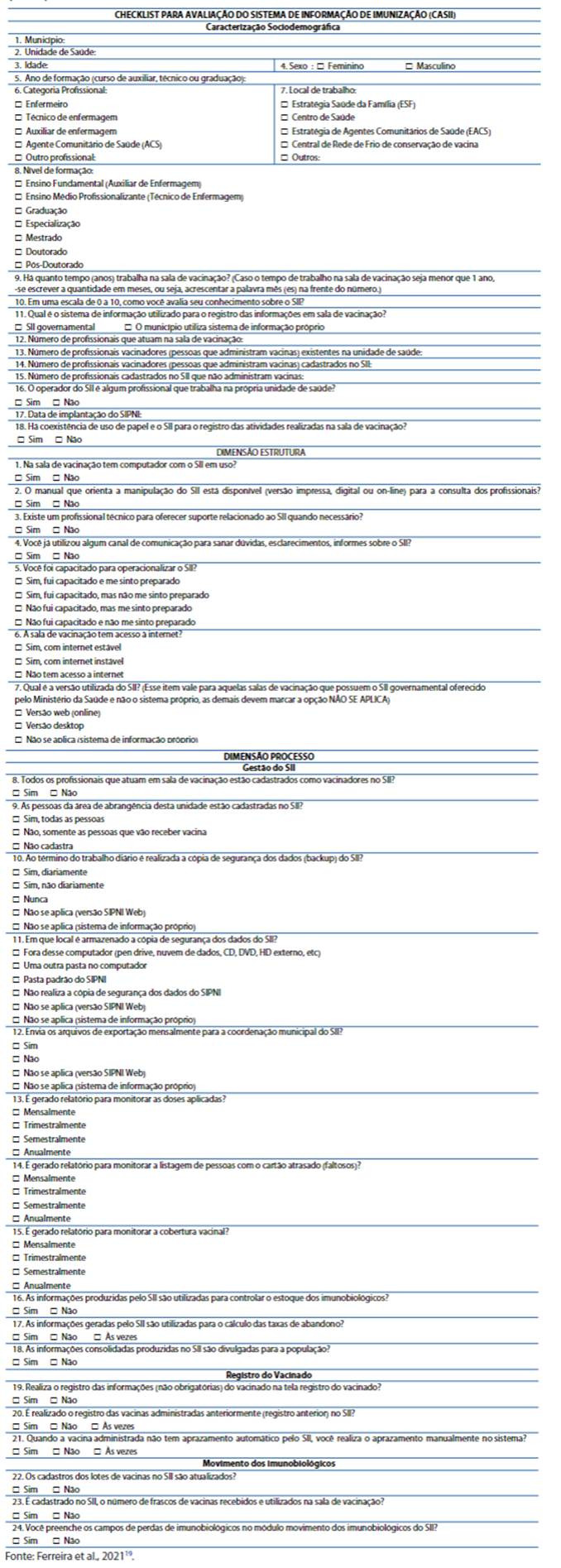
Checklist para Avaliação do Sistema de Informação de Imunização (CASII)
